# Early Sequential Risk Stratification Assessment to Optimize Fluid Dosing, CRRT Initiation and Discontinuation in Critically Ill Children with Acute Kidney Injury: Taking Focus 2 Process Article

**Published:** 2020-10-12

**Authors:** Jean-Philippe Roy, Kelli A. Krallman, Rajit K. Basu, Ranjit S. Chima, Lin Fei, Sarah Wilder, Alexandra Schmerge, Bradley Gerhardt, Kaylee Fox, Cassie Kirby, Stuart L. Goldstein

**Affiliations:** 1 Department of Center for Acute Care Nephrology, Cincinnati Children’s Hospital Medical Center, Cincinnati, Ohio, USA; 2 Department of Pediatric Critical Care Medicine, Children’s Healthcare of Atlanta, Atlanta, GA, USA; 3 Department of Critical Care Medicine, Cincinnati Children’s Hospital Medical Center, Cincinnati, Ohio, USA; 4 Department of Biostatistics and Epidemiology, Cincinnati Children’s Hospital Medical Center, Cincinnati, Ohio, USA; 5 Department of Pediatrics, University of Cincinnati College of Medicine, Cincinnati, Ohio, USA

**Keywords:** Acute Kidney Injury (AKI), Renal Angina Index (RAI), NGAL, Furosemide Stress Test (FST), Children

## Abstract

**Background:**

Acute Kidney Injury (AKI) is common in critically ill children and is associated with increased morbidity and mortality. Recognition and management of AKI is often delayed, predisposing patients to risk of clinically significant fluid accumulation (Fluid Overload (FO)). Early recognition and intervention in high risk patients could decrease fluid associated morbidity. We aim to assess an AKI Clinical Decision Algorithm (CDA) using a sequential risk stratification strategy integrating the Renal Angina Index (RAI), urine Neutrophil Gelatinase-Associated Lipocalin (NGAL) and the Furosemide Stress Test (FST) to optimize AKI and FO prediction and management in critically ill children.

**Methods/Design:**

This single center prospective observational cohort study evaluates the AKI CDA in a Pediatric Intensive Care Unit (PICU). Every patient ≥ 3 months old has the risk score RAI calculated automatically at 12 hours of admission. Patients with a RAI ≥ 8 (fulfilling renal angina) have risk further stratified with a urine NGAL and, if positive (NGAL ≥ 150ng/mL), subsequently by their response to a standardized dose of furosemide (namely FST). RAI negative or NGAL negative patients are treated per usual care. FST-responders are managed conservatively, while non-responders receive fluid restrictive strategy and/or continuous renal replacement therapy (CRRT) at 10%-15% of FO. 2100 patients over 3 years will be evaluated to capture 210 patients with severe AKI (KDIGO Stage 2 or 3 AKI), 100 patients with >10% FO, and 50 requiring CRRT. Primary analyses: Standardizing a pediatric FST and assessing prediction accuracy of CDA for severe AKI, FO>10% and CRRT requirement in children. Secondary analyses in patients with AKI: Renal function return to baseline, RRT and mortality within 28 days.

**Discussion:**

This will be the first prospective evaluation of feasibility of AKI CDA, integrating individual prediction tools in one cohesive and comprehensive approach, and its prediction of FO>10% and AKI, as well as the first to standardize the FST in the pediatric population. This will increase knowledge on current AKI prediction tools and provide actionable insight for early interventions in critically ill children based on their level of risk.

## INTRODUCTION

Acute Kidney Injury (AKI) is a clinical syndrome frequently occurring in hospitalized children, especially in the setting of critical illness, and is associated with poor outcomes. A large multinational collaboration showed that AKI occurs in one out of four children admitted to the Pediatric Intensive Care Units (PICU). Furthermore, severe AKI (KDIGO stages 2 and 3) in children and need for Continuous Renal Replacement Therapy (CRRT) is associated with significantly increased risk of mortality [1,2]. Delay in AKI recognition can worsen fluid overload (FO), a factor that have been independently associated with increased mortality, ventilation time and length of stay when exceeding 10%-15% of patient PICU admission weight [3-5].

Serum Creatinine (SCr), routinely used to assess AKI, is a poor biomarker in acute situations. Its rise is delayed, even with severe compromise of kidney function, and lacks sensitivity for renal tissue damage [6]. As a result, it is unable to effectively distinguish high risk patients early in their clinical course. To overcome this in recent years clinicians have turned to clinical prediction tools to better assess risk of AKI. Such tools incorporate AKI risk scores, biomarkers and functional assessment to target early interventions. The Renal Angina Index (RAI) is an AKI risk score used in the PICU with a very good negative predictive value, and is easily usable for screening large populations [7,8]. An increased concentration of urine Neutrophil Gelatinase-Associated Lipocalin (NGAL), a tissue injury biomarker, precedes the rise in serum creatinine based AKI, and has moderately good predictive performance for AKI [9-11]. The Furosemide Stress Test (FST) predicts the progression of AKI and the need for RRT based on the tubular response to a standardized dose of furosemide [12,13]. Each strategy has its own set of strengths and weaknesses, but we have previously shown in a small pilot study that the RAI and NGAL can be used in complementary and sequential manner to improve AKI prediction. The combination of these two increases their predictive ability, with RAI+/NGAL+(NGAL ≥ 150ng/ml) increasing the area under the curve receiver-operating characteristic (ROC) from 0.80 to 0.97 for severe AKI prediction compared to RAI alone [14]. This current large scale prospective study, (Trial in AKI using NGAL and Fluid Overload to optimize CRRT Use, TAKING FOCUS 2) evaluate the feasibility and accuracy of a sequential risk-assessment for AKI prediction in critically ill children using the RAI, urine NGAL and the FST. Our ultimate aim is to determine if this sequential strategy can improve prediction of severe AKI, and provide feasible clinical decision support for fluid management and CRRT initiation.

## METHODS/DESIGN

### Design

This is a prospective, single center, observational study of critically ill children admitted to the PICU.

### Setting

This study will be conducted at Cincinnati Children’s Hospital Medical Center (CCHMC) in a 35 bed tertiary PICU, receiving over 2000 admissions annually, currently using a clinical AKI pathway mirroring the Clinical Decision Algorithm (CDA) explained in this manuscript. Patients with active cardiac problems or post-cardiac surgery and pre-term neonates are admitted to separate units.

### Population

All patients admitted to the PICU fulfilling all inclusion and no exclusion criteria (Table 1).

### Inclusion criteria

≥ 3 months

PICU admission ≥ 48 hours duration

### Exclusion criteria

Baseline CKD Stage IV or V (estimated GFR<30 ml/min/1.73 m^2^)

Active DNR order or the clinical team is not committed to escalating medical care

Acute kidney injury or disease requiring RRT prior to PICU admission

History of kidney transplantation (only for FST)

Known history of allergic reaction to furosemide (only for FST)

Evidence of volume depletion (only for FST)

### Renal angina index

**Figure 1:**
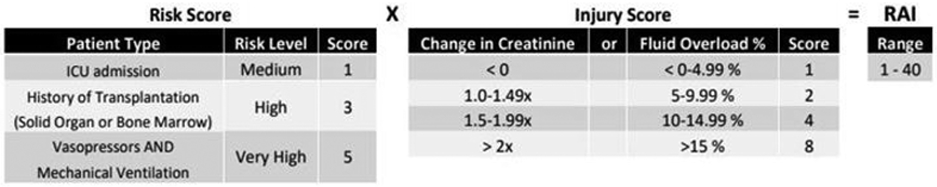
Renal angina index calculation. The composite score is a multiplication the Risk Score and the Injury Score at 12 hours of PICU admission. The Risk Score defaults to 1 for anyone in the PICU, it increases to 3 for transplant recipients (solid organ or bone marrow) and to 5 if the patient is mechanically ventilated and on pressor. The Injury score is attributed a value of 1, 2, 4 or 8 based on the rise of SCr from its baseline or the percentage of FO, whichever value is highest.

We have developed and validated the RAI as a clinical score to assess for AKI risk in critically ill children. It combines patient risk factors of AKI and early signs of kidney dysfunction, giving importance to early mild signs in patients at high risk (Figure 1). Children who “rule in” for renal angina (RAI ≥ 8, RAI+) at 12 hours of ICU admission experience increased Rates of Renal Replacement Therapy (RRT) use, prolonged duration of mechanical ventilation and higher mortality [4,5]. Patients with a RAI<8 (RAI−) are at very low risk of developing persistent severe AKI to day 3 of ICU stay, with a negative predictive value 94%-99% [4,5]. A locally developed computer algorithm extracts data from the electronic health record (EHR, EPIC™, Verona, Wisconsin) to automatically calculate the RAI 12 hours into PICU admission, in eligible patients, and populate the result in the EHR amongst the patient’s lab results. It is recalculated if the patient is returning from the operating room or interventional radiology since their clinical status may have changed. The algorithm identifies a measured SCr value closest to 90 days prior to admission, within a ± 1-month window, as the baseline SCr. If none is found, it imputes a baseline SCr based on the most recent patient height, within the last year, by assuming an eGFR of 120 mL/min/1.73 m^2^, as validated in the pediatric literature [1,15]. If no height is available, it uses patient age to impute a baseline SCr with a similar accuracy, as we have previously validated for this project [16].

## NGAL

Biomarkers of renal tissue injury allow clinicians to differentiate between functional changes in Glomerular Filtration Rate (GFR) and presence of damage, as exemplified by urine marker of tubular damage NGAL.

A meta-analysis of its use in a myriad of pediatric critical settings reported a combined sensitivity and specificity for AKI diagnosis of 0.73 and 0.93 with a diagnostic OR of 43 (95% CI 16–115) [11]. It rises early in the presence of renal injury, even before changes in GFR, and can identify subclinical AKI, defined as renal damage without GFR change associated with a worse outcome compared to no AKI [9-11].

At CCHMC’s clinical lab, urine NGAL is measured using the NGAL Test™ assay (Bioporto Diagnostic, Denmark). During the study period, the analyzer initially was Siemens Dimension Vista 3000t, using the nephelometric module, however it was changed during July 2019 to a turbidometric method using the Siemens Atellica. Values were initially reported between <25 to 15000 ng/ml by manual dilution, but was changed to <50 to 18000 ng/mL by automated dilution with the new analyzer. NGAL levels were comparable between the 2 analyzers. The NGAL result is available within two hours and automatically populated in the EHR.

### Furosemide stress test

The FST, a functional assessment of tubular reserve with a single standardized dose of furosemide, has shown to predict worsening AKI and need for RRT in critically ill adults with an accuracy that exceeds most biomarkers used alone when urine output fails to increase above 200 ml over two hours following a single dose of intravenous furosemide (1 mg/kg in furosemide-naïve vs. 1.5 mg/kg in those previously exposed) [12,13]. In adults, furosemide exposure within the 7 days prior to the FST was used to label a patient as previously exposed, since chronic exposure is frequent in adults and a longer period is needed to reset the distal tubule compensation [17]. FST is contraindicated in patients with evidence of intravascular depletion or with known allergy to furosemide. While promising, the FST has not yet been prospectively evaluated or standardized in children. Hence a working definition of FST was used for the CDA while data are prospectively collected. Furosemide dosing was adapted from the adult study, but the time to last exposure was reduced to 24 hours since chronic furosemide exposure is rare in children without cardiac disease. The threshold of urine output response has been modified to an increase of 1 ml/kg/hr over 2 or 6 hours, since all patients do not have urinary catheters in place for timely measurement. This threshold is a working definition for response and will be reevaluated throughout the course of this study if a better threshold becomes apparent.

### Clinical decision algorithm

To optimize early detection and management of AKI, a CDA based on the current literature was established in January 2018 in the PICU of the study institution as collaboration between the critical care and nephrology providers. This CDA consists of a sequential risk stratification evaluation meant to identify patients at high risk of severe AKI and RRT requirement early in their clinical course and offer guidance on management.

**Figure 2:**
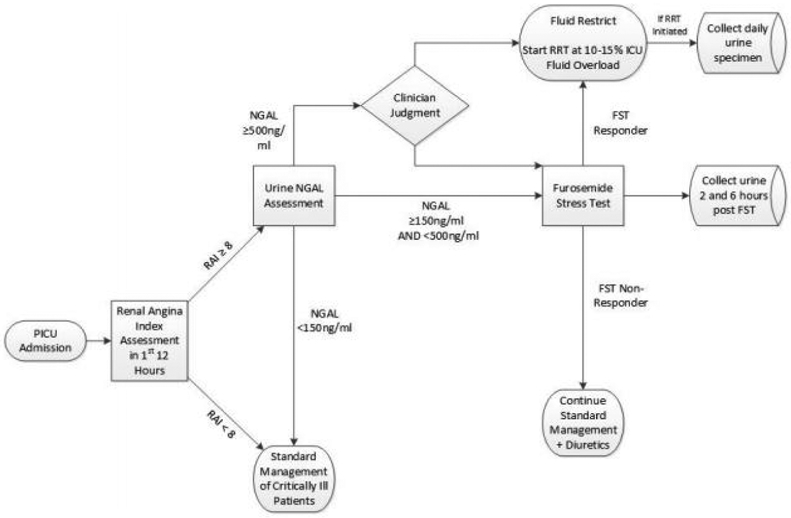
Clinical AKI pathway flow diagram. The clinical support algorithm suggests that patients at low risk, RAI− and RAI+/NGAL−, receive standard management per PICU. Patient at high risk, RAI+/NGAL+, with NGAL 150-500 ng/ml can have their risk further stratify with an FST, unless contraindicated, while those >500 ng/ml can either have an FST or initiate RRT if there is an emergent indication or if it is deemed better/urgent by the primary team. FST-responders have a lower risk of requiring RRT, as such, management with diuretic and fluid restriction is suggested, while FST-nonresponses are likely to fail diuretic management and an initiation of RRT is suggested if FO >10% cannot be prevented by fluid restriction alone.

Every PICU patient fulfilling criteria have a RAI automatically generated 12 hours from admission or return from a surgical or interventional radiology procedure. All admissions have a conditional order for a urine NGAL that automatically releases when a RAI ≥ 8 is populated in their EHR, and a best practice alert notifies the bedside nurse to collect a urine sample. The RAI and NGAL status are intended to be available for clinical decision making within 24 hours of PICU admission (Figure 2). The CDA suggests that patients at low risk, RAI− and RAI+/NGAL−, receive standard PICU management. Patients at high risk, RAI+ with NGAL 150-500 ng/ml, can have their risk further stratified with a FST, unless contraindicated, while RAI+ with NGAL ≥ 500 ng/ml can either have a FST or, based on clinician judgment, RRT initiated. FST responders have a lower risk of requiring RRT, and as such, management with diuretics and fluid restriction is suggested, while FST non-responders are likely to fail diuretic management and RRT initiation is suggested if fluid overload (FO) above 10%-15% cannot be prevented.

### Urine sampling

Convenience urine samples are collected in patients with an indwelling urinary catheter during FST or daily while on CRRT. These samples are frozen for potential future biomarker analyses.

### Data collection

#### Baseline:

Data collection is structured based on individual PICU admissions or encounters, with an individual patient possibly counting towards multiple PICU admissions. Within each PICU admission, baseline data collection includes patient demographics, ICU admission diagnoses using broad pathophysiologic categorization [1], co-morbidities, and baseline kidney function (SCr and GFR).

#### Clinical decision algorithm:

The results of the RAI, NGAL, and FST assessments, as described above, are documented as well as the dates and times of each assessment. RAI results in the medical record are verified through data collection of SCr, FO, organ transplant status, and the use of invasive ventilation and vasopressors during the first 12 hours of PICU admission. For PICU admissions found to be RAI+ without a urine NGAL result, the reason for the missing NGAL is also recorded. AKI status as defined by KDIGO is documented at the time of FST assessment, as well as hourly urine output in the 6 hours prior and 6 hours after the FST [18].

#### Daily:

Data collected daily through study day 7 include fluid balance, laboratory results of kidney function, need for RRT, and the use of Extra Corporeal Membrane Oxygenation (ECMO). Fluid balance includes all fluid intake, all fluid output, and separate reporting of urinary output per day. Using ICU admission weight, daily fluid balance is used to calculate a cumulative FO [5]. FO is calculated as the difference between fluid intake and output in liters divided by PICU admission weight in kilograms and reported in percentage. This calculation excludes the initial fluid resuscitation needed before PICU admission.


FO=(Fluid in (liters)−Fluid Out (liters)/(PICU admission weight(kg)×100%[5]

#### Outcomes:

Patients are followed through Day 28 after PICU admission. At PICU discharge, hospital discharge, and Day 28, each patient is assessed for return to baseline of SCr, FO, need for RRT, and mortality. In addition, at PICU discharge, days on ventilator and use of ECMO are recorded.

FO in the PICU is an outcome of interest since it has emerged as a potentially modifiable risk factor for worse prognosis in patients with AKI. Higher FO is associated with a longer PICU stay, longer ventilation time, and increased risk of mortality in patients requiring CRRT when FO is above 10% at initiation. This risk linearly increases with worsening FO, reaching OR of 8.5 in children with FO >20% [3,5].

### Waiver of consent

A waiver of consent was granted for this study by the IRB of record, because the research presents no more than minimal risk and a waiver would not adversely affect the welfare of PICU patients. In addition, given the nature of AKI, its incidence and the number of patients admitted to the PICU, an evaluation of the whole population is necessary to obtain accurate epidemiologic data and evaluate the timeliness a sequential risk stratification approach. With the relatively high mortality rates of pediatric patients requiring CRRT, requiring consent could present a large exclusion bias, especially for obtaining urine specimen from these patients.

### Co-enrollment

Patients enrolled in TAKING FOCUS 2 may be enrolled in other studies. At the time of study initiation, there was no interventional study aimed at preventing AKI at the study institution. However, in the PICU, there was one interventional device study aimed at improving patient survival, enrolling a subset of patients on CRRT [19]. An overlap of less than 5% of patients requiring CRRT is expected between these two studies.

### Urine sampling

Convenience urine samples are collected in patients with an indwelling urinary catheter during FST or daily while on CRRT. These samples are frozen for potential future biomarker analyses.

### Primary and secondary outcomes

This study’s primary focus is to evaluate the feasibility and accuracy of a CDA, based on sequential risk stratification for AKI using RAI-NGAL-FST, and its accuracy for predicting FO and AKI. Secondary aims include the evaluation and standardization of a pediatric FST. In addition, the association of RAI, NGAL, FST, and their combinations, will be evaluated for their impact on FO and major adverse kidney events (MAKE, including return to baseline renal function, RRT dependence and mortality) at PICU discharge, 7 days and 28 days. The association between FO>10% and different comorbidities and mortality will also be explored.

### Sample size

We designed this project to utilize RAI-NGAL-FST to direct care for patients at each step along the CDA. Once patients have developed standard indications for CRRT, clinicians are allowed flexibility in timing within evidence-based constraints to initiate treatment based on FO. Standard statistical sample size power analysis cannot be performed as we are aiming to evaluate a clinical protocol. The CCHMC PICU admits over 2000 patients annually, with approximately 700 admissions lasting more than 48 hours. Data from the CHERUB [20] and AWARE [1] studies demonstrate that severe AKI occurs in 10%-13% of these admissions. Data from the AWARE study show that 24% of all patients admitted to the PICU will develop 10% FO in 7 days. This study aims to screen 2,100 patients over 3 years with an expectation that 210 will develop severe AKI, 150 will have a urine NGAL >150 ng/ml, 100 patients will develop >10% FO, and 50 of patients will require CRRT. We expect to detect a difference if RAI+ sensitivity of 70% from 60%, at 0.05 significance level and 85% power. Also, considering the specificity of RAI-prediction, with this sample size, we can detect a difference in specificity from 90% to 85% at very high power (>95%).

### Analyses

Epidemiologic data and the timelines of the CDA will be presented as a descriptive model. The incidence of AKI will be reported and the cohort will be stratified into 4 subpopulations based on KDIGO staging: No AKI, AKI stage 1, 2 and 3. Adjusted and unadjusted survival analysis with cox regression models will be used to compare mortality and need for RRT between groups. Groups based on level of FO will also be analyzed in the same way to compare mortality. Each diagnostic test as well as the combined algorithm will be evaluated for sensitivity, specificity, positive and negative predictive value, likelihood ratios and ROC. Youden’s J index will be used to guide selection of an optimal urine output rate to define responders for the FST. Association between diagnostic tests fulfillment and clinical outcomes will be assessed using Chi-square, Fisher’s test or Pearson correlation coefficient depending on the appropriate situation. Those analyses will consider all RAIs, since a single patient maybe have more than one RAI generated during the study period. The feasibility analysis of the clinical decision support algorithm will be defined as the ability to successfully conduct all three tests in >90% of eligible patients within 48 hours of PICU admission, with the RAI and NGAL results available within 24 hours.

## DISCUSSION

Children with AKI are at increased risk of mortality or long term renal sequela [21-27]. While the awareness for pediatric AKI has improved over the years, relying on SCr alone for AKI prediction and ascertainment is bound to introduce delay in recognizing and treating the condition. If high risk patients were identified early in their clinical course, nephrotoxic exposure and FO could be partially avoided. Yet, there is a paucity of large prospective studies analyzing the diagnostic and prognostic accuracy of those new riskstratifying tests in children.

Obtaining data on the feasibility and timeliness of a clinical decision support algorithm will prove that the combination of non-invasive, or minimally invasive, diagnostic testing can accurately predict, early in a clinical course, the risk of AKI and FO in critically ill children. This actionable information will help avoid long escalation of diuretic therapy, modulate the level of care and allow for a thoughtful allocation of time and resources, putting in perspective which patients really need additional care and nephrotoxic avoidance. TAKING FOCUS 2 will be one of the largest studies of AKI in children and will be the first to prospectively evaluate FST in that population.

Although this study is designed to limit bias (e.g. capturing the entirety of the PICU population over a long period of time to eliminate selection bias) it still presents certain limitations. Being an observational study of a clinical protocol, we cannot be ensured that the interventions, like FST and CRRT initiation, are being done according to the established algorithm. While the research team is working in collaboration with both critical care and nephrology providers, their role is to make sure the clinical team is aware of the available information, not to enforce the application of the algorithm.

## CONCLUSION

Hence, some patients may not receive a FST if the providers consider their clinical status to be improving, resulting in a varying amount of data available for the FST over time and across providers. Moreover, RRT requirement is a subjective outcome in studies as its indications can be seen differently by individual providers. Some patients may not be initiated solely on their FO, as suggested by the algorithm, or others may not receive adequate diuretic management for fear of worsening their kidney function, even if the patient exhibits overt FO.

AKI is a clinical syndrome with deleterious impact on patients’ outcomes and prevention with early management is essential. TAKING FOCUS 2 will pave the way to a new era of early and actionable AKI risk recognition in critically ill children.

## Figures and Tables

**Table 1:** Inclusion and exclusion criteria for taking focus 2.

Inclusion criteria	Exclusion criteria
(if all of the following met)	(If any of the following met)
For epidemiology and outcome data collection:	• Baseline CKD stage IV or V*
• ≥ 3 months	• Active DNR order or the clinical team is not committed to escalating medical care
• PICU admission ≥ 48 hours duration	• Acute kidney injury or disease requiring RRT prior to PICU admission
	Only for FST: • History of kidney transplantation • Known history of allergic reaction to furosemide • Evidence of volume depletion

Note:

* estimated GFR <30 ml/min/1.73m^2^

Taking Focus 2: Trial in AKI using NGAL and Fluid Overload to optimize CRRT Use; PICU: Pediatric Intensive Care Unit; CDA: Clinical Decision Algorithm; RAI: Renal Angina Index; NGAL: Neutrophil Gelatinase-associated Lipocalin; CKD: Chronic Kidney Disease; DNR: Do Not Resuscitate; RRT: Renal Replacement Therapy; FST: Furosemide Stress Test
